# Multi‐cohort cerebrospinal fluid proteomics identifies robust molecular signatures for asymptomatic and symptomatic Alzheimer’s disease

**DOI:** 10.1002/alz.084185

**Published:** 2025-01-09

**Authors:** Muhammad Ali, Jigyasha Timsina, Yuanyuan Shen, Anh Do, Lihua Wang, Daniel Western, Ellen Liu, Aleksandra Beric, Gyujin Heo, John P. Budde, Jen Gentsch, Suzanne E. Schindler, John C. Morris, David M. Holtzman, Agustin Ruiz, Ignacio Alvarez, Miquel Aguilar, Pau Pastor, Jarod Rutledge, Hamilton Oh, Edward N. Wilson, Yann Le Guen, Rana Rehan Khalid, Alzheimer's Disease Neuroimaging Initiative (ADNI), Knight Alzheimer Disease Research Center (Knight ADRC), Fundació ACE Alzheimer Center Barcelona (FACE), Stanford Alzheimer Disease Research Center (Stanford ADRC), Chloe Robins, David J. Pulford, Laura Ibanez, Tony Wyss‐Coray, Yun Ju Sung, Carlos Cruchaga

**Affiliations:** ^1^ NeuroGenomics & Informatics Center, St. Louis, MO USA; ^2^ Department of Psychiatry, Washington University School of Medicine, St. Louis, MO USA; ^3^ NeuroGenomics & Informatics Center, Washington University School of Medicine, St. Louis, MO USA; ^4^ Washington University in St. Louis, School of Medicine, St. Louis, MO USA; ^5^ Neurogenomics & Informatics Center, St. Louis, MO USA; ^6^ Neurogenomics & Informatics Center, Saint Louis, MO USA; ^7^ Washington University School of Medicine, Saint Louis, MO USA; ^8^ Hope Center for Neurological Disorders, St. Louis, MO USA; ^9^ Washington University in St. Louis School of Medicine, St. Louis, MO USA; ^10^ Knight Alzheimer Disease Research Center, St Louis, MO USA; ^11^ Washington University in St. Louis, St. Louis, MO USA; ^12^ Department of Neurology, Washington University School of Medicine, St. Louis, MO USA; ^13^ The Charles F. and Joanne Knight Alzheimer Disease Research Center, St. Louis, MO USA; ^14^ Washington University School of Medicine in St. Louis, St. Louis, MO USA; ^15^ Washington University School of Medicine in St Louis, St. Louis, MO USA; ^16^ Knight Alzheimer Disease Research Center, St. Louis, MO USA; ^17^ Networking Research Center on Neurodegenerative Diseases (CIBERNED), Instituto de Salud Carlos III, Madrid Spain; ^18^ Research Center and Memory Clinic, Fundació ACE Institut Català de Neurociències Aplicades ‐ Universitat Internacional de Catalunya (UIC), Barcelona Spain; ^19^ Research Center and Memory Clinic, ACE Alzheimer Center Barcelona, Universitat Internacional de Catalunya, Barcelona Spain; ^20^ Fundació Docència i Recerca Mútua de Terrassa, Neurology Research Unit, Terrassa Spain; ^21^ Unit of Neurodegenerative diseases, University Hospital Germans Trias i Pujol, Badalona, Barcelona Spain; ^22^ Wu Tsai Neurosciences Institute, Stanford University, Stanford, CA USA; ^23^ Stanford University School of Medicine, Stanford, CA USA; ^24^ Stanford University, School of Medicine, Stanford, CA USA; ^25^ National Centre for Bioinformatics, Quaid‐i‐Azam University, Islamabad, Punjab Pakistan; ^26^ Laboratory of Neuro Imaging (LONI), University of Southern California, Los Angeles, CA USA; ^27^ Knight Alzheimer's Disease Research Center, Washington University, St. Louis, MO USA; ^28^ Fundació ACE Alzheimer Center, Barcelona Spain; ^29^ Stanford Alzheimer Disease Research CenterStanford Alzheimer Disease Research Center, Stanford, CA USA; ^30^ Human Genetics & Genomics, Research Technologies, GSK, Upper Providence, PA USA; ^31^ Human Genetics & Genomics, Research Technologies, GSK, Stevenage, Hertfordshire UK

## Abstract

**Background:**

Changes in Amyloid‐β (A) and hyperphosphorylated Tau (T) in the brain and cerebrospinal fluid (CSF) precedes AD symptoms, making the CSF proteome a potential avenue to understand disease pathophysiology and facilitate reliable diagnostics and therapies.

**Method:**

We used the Somascan assay for measuring the protein levels of 7,029 analytes in CSF of 2,286 participants from four different cohorts. We employed a three‐stage analytical approach (discovery, replication, and meta‐analysis). Firstly, discovery was performed in 1,170 samples from the Knight‐ADRC and FACE cohorts (stage1) using the ATN framework (A‐T‐ =680 and A+T+ =490). Secondly, proteins that passed FDR correction in the stage1 were further tested in 593 individuals (A‐T‐ =235 and A+T+ =358) from the ADNI and Barcelona‐1 replication cohorts (stage2). Lastly, the proteins that passed Bonferroni corrections on the meta‐analysis of stage 1 and 2 were further utilized for creating AD prediction models, conducting pathway enrichment, disease progression, and protein‐protein interaction (PPI) network analyses to gain mechanistic insights into AD pathophysiology.

**Result:**

We identified 2,173 proteins dysregulated in AD, that were further validated in a third totally independent cohort (Stanford‐ADRC). Using machine learning, we identified a distinctive signature of 11 proteins with robust and high predictive capability for AD (AUC = 0.97–0.99) in three independent cohorts (stage1, stage2, and Stanford‐ADRC). The identified proteomic signature was unique to AD and showed no predictive power for other dementias such as frontotemporal dementia (AUC=0.61), dementia Lewy body (AUC=0.73), or Parkinson’s disease (AUC=0.57). The developed proteomic signature displayed significant association with disease progression (β=0.35, p=2.1×10^‐04^) and individual’s probability of not developing AD (p=2.2×10^‐58^). The associated proteins cluster in four different protein pseudo‐trajectory groups spanning the AD continuum and were enriched in specific pathways including neuronal death, apoptosis and tau phosphorylation (early‐stages), microglia dysregulation and endolysosomal dysfunction (mid‐stages), and late microglia‐neuron crosstalk (late‐stages).

**Conclusion:**

Our findings show the promising potential of AD CSF proteomics in developing reliable and robust AD prediction model. We identified proteins and biological pathways that are compromised in AD, thereby, increasing our understanding of AD biology and facilitating the development of effective therapies targeted at earliest molecular triggers of AD.

